# Acute Coronary Artery Occlusion during Transcatheter Aortic Valve Replacement in a Patient with an Anomalous Left Circumflex Coronary Artery

**DOI:** 10.1155/2022/6257367

**Published:** 2022-07-08

**Authors:** Rongfeng Xu, Jiandong Ding, Lijuan Chen, Yi Feng, Genshan Ma

**Affiliations:** Department of Cardiology, Zhongda Hospital, Medical School of Southeast University, Nanjing, 210009 Jiangsu, China

## Abstract

**Background:**

Acute coronary artery occlusion (CAO) during transcatheter aortic valve replacement (TAVR) is a rare but life-threatening complication during the procedure; there were a few case reports about an anomalous LCX during perioperative period. We report a case of successful coronary protection using the chimney stenting technique in a patient with a severely calcified aortic valve and an anomalous LCX. *Case Summary*. A 75-year-old man was found an anomalous left circumflex coronary artery (LCX) originating from the right coronary cusp with severely calcified aortic valve stenosis requiring TAVR. When a self-expanding aortic valve was deployed, we found flow compromise in the right coronary system and circumflex to TIMI-0 flow. By using the chimney stenting technique, we rapidly planted 2 stents from the proximal CX branch to the sinotubular junction and the coronary flow was maintained.

**Conclusion:**

Chimney stenting protection as a bailout technique is safe and feasible and should be considered in patients deemed to be at high risk of coronary flow compromise, especially with an anomalous LCX.

## 1. Introduction

Although traditional surgical aortic valve replacement (SAVR) has been the mainstream treatment method for symptomatic severe aortic stenosis, transcatheter aortic valve replacement (TAVR) is becoming a standard-of-care procedure for those patients who would be at high or intermediate risk for cardiac surgery. Acute coronary artery occlusion (CAO) during TAVR is a rare (<1%) but life-threatening complication in contemporary practice, and specific subsets of patients remain at risk [1]. Displacement of the calcified native valve leaflet over the coronary ostium and direct occlusion of the coronary ostium by the covered skirt of the transcatheter aortic prosthesis are the usual causes of CAO. This phenomenon is associated with anatomical factors, including lower-lying coronary ostia and shallow sinuses of Valsalva (SOVs), and especially valve-in-valve (VIV) for surgical bioprostheses [2]. Furthermore, an anomalous coronary artery may be an unusual cause of CAO [3]. There are only a few reports of TAVR in the context of an anomalous LCX [4]. Coronary protection during TAVR is a preemptive technique recommended in certain cases to avoid this complication. As an important bailout technique, the “chimney” stenting technique is performed during or after TAVR if coronary blood flow is compromised [5]. We report a case of successful coronary protection by using the chimney stenting technique in a patient with a severely calcified aortic valve and an anomalous LCX.

## 2. Case Presentation

**Figure 1:**
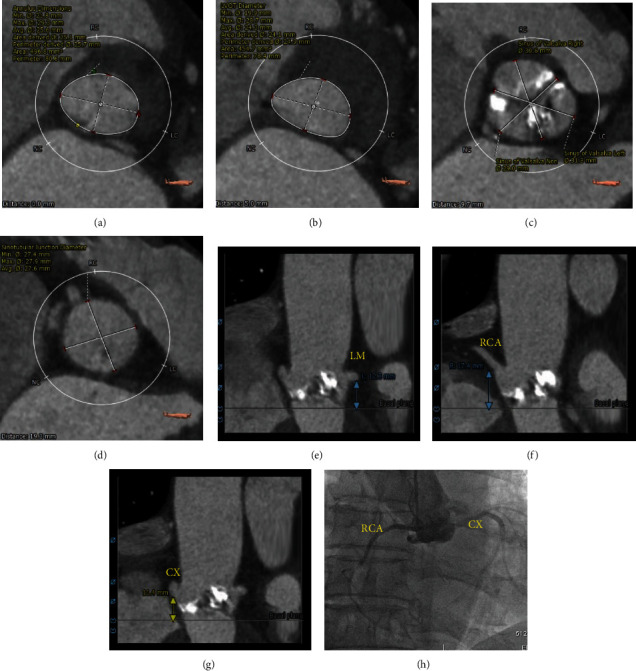
Cardiac computed tomography angiography showed severely calcified aortic leaflets with anatomical abnormalities of the right coronary system, which revealed a circumflex branch originating from the right coronary sinus, as shown by CAG.

**Figure 2:**
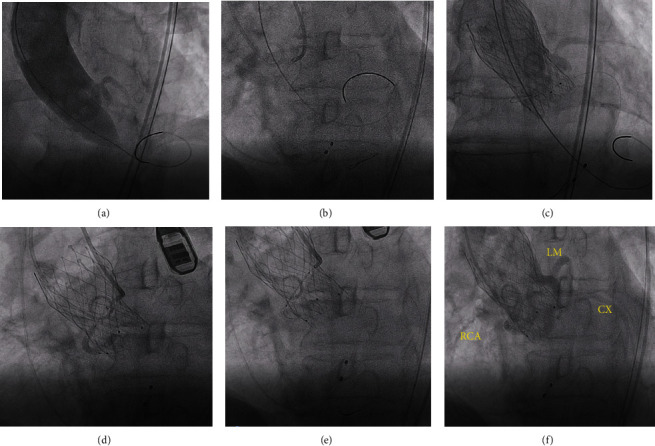
Balloon aortic valvuloplasty and simultaneous aortic root injection showing decreased coronary flow to TIMI-0 in the RCA and CX. One guidewire was advanced to the RCA and CX with a 2.0∗20 mm semicompliant balloon. There was flow compromise in the right coronary system and CX to TIMI-0 flow after TAVR, followed by deployment of two stents from the proximal CX branch to the sinotubular junction. The flow of the right coronary artery and CX was retained, and the elevated ST segment in the EKG was reversed.

**Figure 3:**
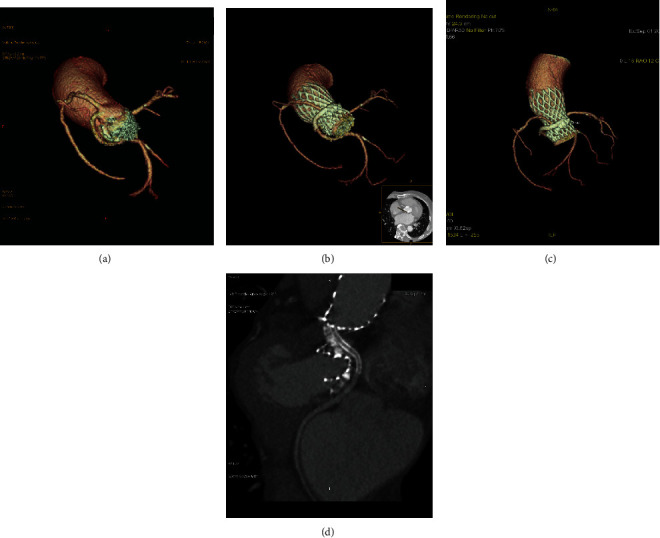
Six-month follow-up results after TAVR. (a) Cardiac computed tomography angiography showed a circumflex branch originating from right coronary sinus before TAVR. (b–d) Six-month follow-up after TAVR. CTA of the aortic and coronary regions suggested unobstructed coronary flow of the CX.

A 75-year-old man was admitted to our hospital with a history of active dyspnea for 1 year and no coronary heart disease, hypertension, or diabetes. He had been smoking for 40 years, with 10 cigarettes per day. Physical exam revealed a grade 5 crescendo-decrescendo murmur without elevated JVP and lower extremity edema. Laboratory findings were pertinent for creatinine NT pro-BNP of 2320 pg/ml and TNI 0.023 ng/ml. The rest of his physical exam and laboratory was normal. Electrocardiogram showed a normal sinus rhythm with a known right bundle branch block. Echocardiogram demonstrated severe aortic stenosis with a mean aortic valve pressure gradient of 100.5 mmHg and peak velocity of 5.60 m/s, with preserved systolic function. The patient received intravenous diuretics with some clinical improvement. He was seen and evaluated by a cardiothoracic surgeon for evaluation of aortic valve replacement but deemed intermediate risk for SAVR, with an estimated surgical mortality risk of 4.15% according to the Society of Thoracic Surgeons score. Cardiac computed tomography angiography was performed as part of the TAVR evaluation (Figures 1(a)–1(d)) and showed severely calcified aortic leaflets with an anomalous LCX, which revealed a circumflex branch originating from the right SOV. The ostial heights of the left and right coronaries were 12.3 mm and 17.4 mm, respectively (Figures 1(e) and 1(f)), while the ostial height of the circumflex artery was 11.4 mm (Figure 1(g)). Coronary angiography revealed normal left and right coronary arteries and an anomalous circumflex coronary artery without stenosis (Figure 1(h)). Special attention was given to right coronary leaflet calcification and anatomical abnormalities of the circumflex. The decision was then made to proceed with TAVR utilizing the coronary protection technique during the procedure. TAVR was then undertaken from the right femoral artery through a 19F arterial sheath. With rapid ventricular pacing over a temporary transvenous pacing wire (180 bpm), balloon aortic valvuloplasty using a 22 × 40 mm balloon (VENUS MEDTECH, Hangzhou, China) with simultaneous root aortography was performed. There was flow compromise in the right coronary artery and circumflex to TIMI-0 flow (Figure 2(a)). To perform the coronary protection technique to avoid CAO, a 6F JR-4 guide catheter inserted through a transradial access was used to engage the anomalous LCX, and a guiding wire of 0.014 inches was advanced to the CX arteries with a 2.0 × 20 mm semicompliant balloon (Figure 2(b)). A 26 mm Venus-A self-expanding valve (VENUS MEDTECH, Hangzhou, China) with rapid ventricular pacing was then deployed. The position of the valve and function were confirmed by aortography and transesophageal echocardiography (TEE). We found flow compromise in the right coronary system and circumflex to TIMI-0 flow (Figure 2(c)), and the ST segments in the EKG were elevated. One drug-eluting stent (2.75 × 23 mm) was deployed from the proximal CX branch to the ostium, followed by deployment of another drug-eluting stent (3.0 × 18 mm) from the ostium of CX to the sinotubular junction through Guidezilla (Figures 2(d) and 2(e)). The flow of the right coronary artery and circumflex was retained, and the elevated ST segment in the EKG was reversed (Figure 2(f)). The patient tolerated the procedure well without complications, recovered uneventfully, and was discharged 7 days after the procedure without complications. At the 30-day follow-up, he had notable improvement of symptoms and physical activity, with a change to NYHA class I from class III. Six months after being discharged from our hospital, CTA of coronary artery suggested unobstructed coronary flow of the CX (Figures 3(b)–3(d)).

## 3. Discussion

TAVR is an acceptable and effective alternative to SAVR in high-risk or intermediate-risk patients. However, a rare but devastating complication is coronary ostial obstruction during the TAVR procedure. There are some key predictors of CAO, including low coronary ostia, inadequate SOV width, and, in the context of VIV procedures, surgical bioprostheses with externally mounted leaflets or a short virtual transcatheter valve-to-coronary ostium (VTC) distance [6, 7]. An anomalous coronary artery can be a predictor of CAO. In some special patients at high risk of CAO during TAVR procedure, upfront coronary artery protection can be provided by positioning a coronary guidewire, balloon, undeployed stent, or guide extension in the artery [8, 9]. Recent research has reported that chimney stenting is infrequently performed in modern TAVR practice (approximately 0.5% of overall TAVR cases), and this technique is not only performed for the acute treatment of total occlusion of coronary flow but also applied when imaging reveals partial obstruction of the coronary ostium or reduced coronary blood flow and an evolution to complete CAO is anticipated [5]. Thorough evaluation of each case prior to TAVR using coronary angiography, CT scanning, and echocardiography enabled us to identify patients we believed to be at increased risk for coronary compromise during the TAVR procedure. The utilization of the chimney stenting protection method helped in the early diagnosis and efficient treatment of coronary compromise during TAVR. The stent is retracted to extend from the proximal portion of the coronary artery cranially, exteriorly, and parallel to the transcatheter heart valve and is deployed to create a channel for coronary perfusion between the displaced leaflets and the aortic wall. In this case, we successfully performed coronary protection by using the chimney stenting technique in anatomical abnormalities of the circumflex system, which originated from the right SOV. When the ostium of the right coronary artery and circumflex artery were occluded by calcified valve cusps, we rapidly planted 2 stents from the proximal CX branch to the sinotubular junction and created a channel for coronary perfusion between the displaced leaflets and the aortic wall; hence, the coronary flow was maintained. Bioprosthetic or native aortic scallop intentional laceration to prevent iatrogenic coronary artery obstruction during TAVR (BASILICA) is a transcatheter procedure performed immediately before TAVR at high risk of coronary artery obstruction, in which the target aortic native or bioprosthetic leaflets are split using focused radiofrequency energy directed by catheters and guide wires [10]. One-year outcome of the BASILICA trial reveals that there was no late stroke, myocardial infarction, or death related to BASILICA, and the mitigation of coronary obstruction remained intact at 1 year, thereby, the BASILICA technique could avoid the long-term complications related to snorkel stenting [11]. In our case, pre-TAVR cardiac CT imaging suggested a relatively high risk for coronary obstruction in right SOV; therefore, it could be a good alternative to perform BASILICA technique before TAVR.

## 4. Conclusions

Chimney stenting protection as a bailout technique is safe and feasible and should be considered in patients deemed to be at high risk of coronary flow compromise, especially with an anomalous LCX.
